# Exploring the Complexity of Real‐World Health Data Record Linkage—An Exemplary Study Linking Cancer Registry and Claims Data

**DOI:** 10.1002/pds.70120

**Published:** 2025-03-25

**Authors:** Nadja Lendle, Bianca Kollhorst, Timm Intemann

**Affiliations:** ^1^ Department of Biometry and Data Management Leibniz‐Institute for Prevention Research and Epidemiology – BIPS Bremen Germany

**Keywords:** administrative healthcare database, data linkage, deterministic linkage, gradient boosting, pharmacoepidemiology, quasi‐identifiers

## Abstract

**Purpose:**

Record linkage based on quasi‐identifiers remains an important approach as not every data source provides a comprehensive unique identifier. In this study, the reasons for the failure of a linkage based on quasi‐identifiers were examined. Furthermore, informed algorithms using information on gold standard links were developed to investigate the potentially achievable linkage quality based on quasi‐identifiers.

**Methods:**

The study population includes patients from an antidiabetic cohort from German claims and colorectal cancer patients from two German cancer registries. Linkage algorithms were applied using information on gold standard links. Informed linkage algorithms based on deterministic linkage, logistic regression, random forests, gradient boosting, and neural networks were derived and compared. Descriptive analyses were performed to identify reasons for the failure of linkage, such as discrepancies between data sources.

**Results:**

A gradient boosting‐based linkage approach performed best, achieving a precision (positive predictive value) of 77%, a recall (sensitivity) of 81%, and an F*‐measure (combining precision and recall) of 64%. Of 641 patients in GePaRD, 8% were not uniquely identifiable using birth year, sex, area of residence, and year and quarter of diagnosis, whereas 33% of 42 817 cancer registry patients were not uniquely identifiable with these quasi‐identifiers.

**Conclusions:**

Linkage of German claims and cancer registry data based on quasi‐identifiers does result in insufficient linkage quality since subjects cannot be uniquely identified. It is advisable to use unique identifiers from a subsample, if available, to derive informed linkage algorithms for the entire sample. In this case, the machine learning technique gradient boosting has been found to outperform other methods.

**Summary**

- Record linkage approaches of the German cancer registry and GePaRD based on a few quasi‐identifiers showed a precision of up to 77%, a recall of up to 81%, and an F*‐measure of 64%.
- The algorithm based on gradient boosting performed best of all applied linkage algorithms.
- Of the considered 641 patients in GePaRD, 8% were not uniquely identified by birth year, sex, area of residence, and year and quarter of diagnosis. With a larger data source, that is, the cancer registries of Bremen and Lower Saxony with 42 817 patients, even 33% were not uniquely identified with these quasi‐identifiers.

## 1 Introduction

Record linkage (RL) of existing data sources is crucial for enhancing pharmacoepidemiologic research opportunities. Collecting data for a specific research purpose is expensive, time‐consuming, and challenging. Sharing and reusing data is therefore recommended [1]. For example, health claims data can be used for pharmacoepidemiological studies [2], but lack information on tumor grades or laboratory values. This missing information can be supplemented by registries, such as cancer registries (CRs).

Possibilities of linkage of such databases are heavily influenced by data protection requirements and linkage infrastructures. Some countries have implemented a unique identifier in health databases to enable efficient RL (e.g., Community Health Index Number in Scotland [3] or the unique civil registration code in Nordic countries [4]). For other data sources, researchers have to rely on quasi‐identifiers. Quasi‐identifiers cannot uniquely identify the entity on their own, only in combination with others, for example, used when linking Dutch Foundation for Pharmaceutical Statistics and Dutch Arthroplasty Register [5], or linking mother and child pairs in either the Hospital Episode Statistics in the UK [6] or in the German Pharmacoepidemiological Research Database (GePaRD) [7].

The validation of such linkages using quasi‐identifiers is complicated as true links are usually unknown or the linkage is performed by a third‐party trust centre [8]. On the other hand, a linkage algorithm using only quasi‐identifiers might be easier in terms of administration and data protection compared to an approach using direct identifiers.

In addition to validation, the derivation of such linkage algorithms is also a challenge. This can be done using either expert knowledge or informative data. The latter is applicable if a unique identifier is available for at least part of the data. In this case, the algorithm is developed using quasi‐identifiers on this subsample and can then be applied to the entire data set. We call such data‐driven algorithm “informed” whereas the algorithm solely based on expert knowledge is called “uniformed”. Proper derivation and validation are important because linkage errors can lead to a reduction of sample size, misclassification, selection bias, and differential linkage, and thus may influence analysis results [9].

To assess the consequences of linkage between CRs and claims data based on quasi‐identifiers, Kollhorst et al. compared linkage results based on quasi‐ and direct identifiers in a study on the risk of glucose‐lowering treatment on colorectal cancer (CC). For this purpose, GePaRD and German CRs were used [10]. In this study, the unique identifier used was the well‐established control numbers, which are derived at CRs [11] and show high accuracy [12]. The uninformed algorithm based on quasi‐identifiers could not replace the unique identifier. Nevertheless, the reasons for the poor performance remained unclear, as did the potential for improvement considering additional data information.

Using the same data, we evaluated uninformed and informed linkage methods based on traditional and machine learning (ML) techniques and determined the linkage quality. Furthermore, we investigated the reasons for the failure of linkage based on quasi‐identifiers. The data set with the given unique identifier has the rare potential to provide answers to these questions based on real‐world data and enables to derive recommendations for the use of unique and quasi‐identifiers for RL.

## 2 Methods

### 2.1 Data Sources

GePaRD is based on claims data from four statutory health insurance providers (SHIs) in Germany and currently includes information on approximately 25 million persons who have been insured with one of the participating providers since 2004 or later. In addition to demographic data, GePaRD contains information on drug dispensations as well as outpatient (i.e., from general practitioners and specialists) and inpatient services and diagnoses. Further information can be found elsewhere [13]. In Germany, informed consent for studies based on claims data is required by law unless obtaining consent appears unacceptable and would bias results, which was the case in this study.

The population‐based CRs of the federal states of Bremen and Lower Saxony have a completeness of reported cancer cases of more than 95% [14]. The CRs collect data on malignant neoplasms of patients living in their geographical catchment area. The data contain personal identifiers, demographics such as area code, sex, month, and year of birth, information about the tumor including stage, site, and extent at the date of diagnosis (DoDx) as well as follow‐up information of the patient including vital status, date, and cause of death.

We used data from the previously published study by Kollhorst et al. [10]. In this previous study, data from an antidiabetic cohort from GePaRD and data of CRs of Bremen and Lower Saxony were linked, first, using an uninformed deterministic algorithm (UDA) based on quasi‐identifiers, and second, using an established probabilistic control number linkage [11] which is the gold standard. The quasi‐identifiers common to both data sources were birth year, sex, area of residence (AoR), DoDx, diagnosis code according to the *International Statistical Classification of Diseases and Related Health Problem* 10th revision; German Modification (ICD10‐GM), and vital status (Data S1: SM1). Due to data privacy, the CR of Lower Saxony could only provide 50% of all cases. Gold standard links with missing information in quasi‐identifiers were excluded here (*N* = 36). Furthermore, the data only contained the quarter of diagnosis.

Number of patients in GePaRD and CRs and their overlap were calculated, that is, the numbers of cases in GePaRD, the number of cases in CRs, and the joint cases.

Additionally, the vital status variable was compared between data sources.

### 2.2 Record Linkage Algorithms

First, a naïve deterministic 1:1 matching on quasi‐identifiers was conducted, where only unique (bijective) links were kept.

Second, we used the UDA by Kollhorst et al. [10] which is based on quasi‐identifiers from CRs and GePaRD data only. Briefly, the UDA matched inpatient CC cases from GePaRD and cases of the CRs on accordant AoR, sex, and birth year and an absolute difference of DoDx of 90 days. To obtain an exact match, links with a concordant four‐digit ICD code were preferably selected before links with a concordant three‐digit ICD code (step 1). If there was no link with matching ICD codes, links with non‐matching ICD codes were selected. Cases with the closest DoDx were selected (step 2). If still more than one possible match was available, one was randomly selected (step 3).

For training of informed algorithms, a dataset with merged data from both sources, including all possible links with an indicator variable for gold standard links, was created. Afterwards, blocking, that is, the reduction of all potential links to those with the same values for all blocking variables, was applied on birth year, sex, AoR, and 90 days of difference between DoDxs, if not stated otherwise.

Third, the informed deterministic algorithm (IDA) was developed from the UDA using the knowledge of gold standard links. For patients from Bremen, the allowed difference between DoDxs was reduced to 50 days. According to the gold standard, there were no links with a difference of more than 50 days (Data S1: SM2). In contrast to the UDA, the IDA also used the vital status: Before step 3, a further step was added: for deceased patients according to GePaRD, cases also deceased according to CRs were preferred over those cases still alive according to CRs. Only persons deceased according to GePaRD were considered, as many patients alive according to GePaRD were deceased according to the CR data (Table 1).

**TABLE 1:** Accordance of vital status according to GePaRD and cancer registries (CRs) for gold standard links.

Vital status according to GePaRD	Vital status in CRs according to gold standard linkage	Count of gold standard links	Proportion of gold standard links
alive	alive	123	59%
died	died	69	33%
alive	died	15	7%
died	alive	3	1%
Total gold standard links		210	100%

Fourth, logistic regression and the ML method neural networks (according to Keydana [15]) and the tree‐based methods gradient boosting [16] and random forests [17] were used to derive linkage algorithms (R‐packages stats [18], torch [19], xgboost [20], ranger [21] were used respectively). Predictors used are CR (Bremen/Lower Saxony), vital status, ICD10 code (three‐ and four‐digit), and DoDx (depending on the methods either agreement of or the information itself from both sources). The logistic regression model predicted a match probability using additionally an interaction term for CR and (absolute) difference between DoDxs in days. A grid search was used in order to determine a probability threshold to divide between links and no links. Additionally, hyperparameters were optimized regarding precision using another grid search for all methods except logistic regression (Data S1: SM3). Each GePaRD case was then assigned to the case from CRs with the highest probability. In turn, for each linked case of CRs, the GePaRD case with the highest probability was chosen. GePaRD cases that ‘lost’ their link could be linked to their second‐best possible link if that one was not yet linked (Data S1: SM4).

Fifth, probabilistic RL was applied using blocking on birth year, sex, and AoR, and match weights were calculated based on gold standards links (R‐package RecordLinkage [22]). Brief descriptions for all algorithms can be found in Data S1: SM5.

Algorithms were compared using precision, recall and F*‐measure. Precision (i.e., positive predictive value) is defined as $P=\frac{\text{number of true positives}}{\;\text{number of predicted matches}}$ and recall (i.e., sensitivity) as $R=\frac{\text{number of true positives}}{\text{number of gold}-\text{standard matches}}$ [23]. The F*‐measure which indicates the proportion of true positives to number of either predicted or gold standard matches and is calculated as $F^{*}=\mathrm{PR}/\left(P+R-\mathrm{PR}\right)$ [24].

### 2.3 Reasons for Failure of the Linkage

For links from the gold standard, quasi‐identifiers were compared between data sources to identify potential synonym errors.

Ideally, each combination of quasi‐identifiers should be unique within each database. Duplications of these combinations would lead to more than one possible link per case (i.e., homonym error). For this purpose, first only the birth year was considered, and in each subsequent step, a single variable (AoR, sex, and year and quarter of diagnosis) was added. After each step, the proportion of unique entries was calculated. Additionally, the number of entries with identical quasi‐identifiers was counted both per data source and combined. Furthermore, vital status was added to analyze its impact as a linkage variable.

## 3 Results

### 3.1 Data Sources

**FIGURE 1:**
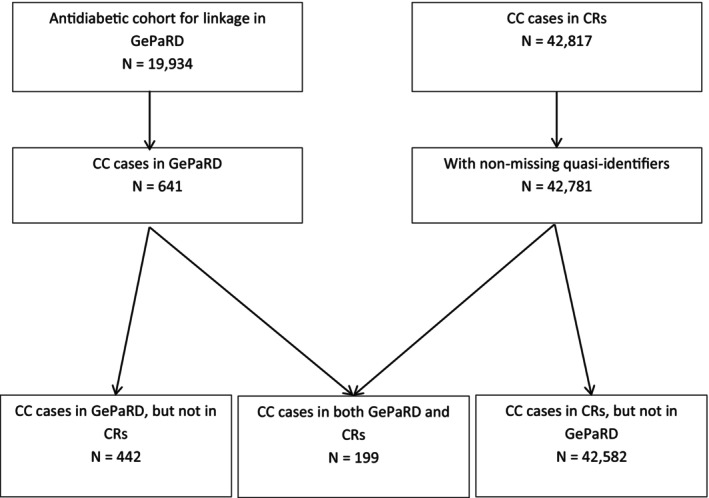
Dataflow for study population based on GePaRD, cancer registries (CRs) and their common gold standard links. CC: colorectal cancer.

Overall, 199 gold standard links with confirmed CC in both databases were found using control number linkage (Figure 1). For 189 out of 363 inpatient GePaRD cases, a gold standard link existed (Data S1: SM6). For the 278 patients with an outpatient diagnosis, there were only 10 gold standard matches. For 11 gold standard links, there was no diagnosis of CC recorded in GePaRD. Six of those patients changed SHI before diagnosis, so this information cannot be available in GePaRD.

In total, 21 links were excluded from linkage due to inconsistencies. For a further 365 cases, the quarter of diagnosis was missing, which greatly reduces the chances of successful linkage.

In about 8% of the gold standard matches, there were inconsistencies between GePaRD and CRs concerning vital status (Table 1). Most of those cases were stored as alive in GePaRD but as deceased according to CRs.

### 3.2 Record Linkage Algorithms

The UDA reached a precision of 54% and a recall of 76%, which was outperformed by the IDA by 4 and 3 percentage points, respectively (Table 2). The naïve deterministic 1:1 matching algorithm resulted in the least links but showed a relatively high precision (59%). Gradient boosting performed best of all methods, with a precision of 77% and recall of 81%. Due to the few matching variables, probabilistic linkage failed since it resulted in very little variation in match weights (Data S1: SM7).

**TABLE 2:** Comparison of different linkage algorithms by key measures.

	UDA	IDA	Naïve deterministic 1:1 matching	Logistic regression	Gradient boosting	Random forest	Neural network
True positives	155	161	56	155	164	147	94
Mismatches	134	119	39	91	49	102	192
Total links	289	280	95	246	213	249	286
Precision	54%	58%	59%	63%	**77%**	59%	33%
Recall	76%	79%	28%	76%	**81%**	72%	46%
F*‐measure	46%	50%	23%	53%	**65%**	48%	24%

*Note:* Two kinds of gold standard links were excluded from those calculations: cases missing quasi‐identifiers and cases with diagnosis after change of SHI. Best results are marked in bold.

Abbreviations: IDA: informed deterministic algorithm, UDA: uninformed deterministic algorithm by Kollhorst et al. [10].

### 3.3 Reasons for Failure of the Linkage

There were inconsistencies between GePaRD and CRs (Table 3). For one gold standard link, sex information differed between GePaRD and CRs. GePaRD used the AoR at the time of data delivery (2017) which differed from those in CRs (at time of diagnosis) in six cases. In 13 cases, the difference between DoDxs exceeded 90 days. The usage of blocking on this quasi‐identifier prevented the successful linkage of those cases. For another 35 cases, there was no accordance of the ICD10 codes (four‐digit) in GePaRD and CRs. Even the three‐digit code differed in four of these cases. While these links might be found, the chances of a mismatch increase.

**TABLE 3:** Distribution of reasons for disaccording data between GePaRD and cancer registries (CRs) for gold standard links.

Cases with missing data (*N*)	Cases with nonconforming data (*N*)					
CR: AoR, birth year, sex	sex	AoR*	More than 90 days between diagnoses	GePaRD: No inpatient diagnosis	ICD10 codes	
					4‐digit	3‐digit
36	1	6	13	11	35	4

*Note:* AoR*: GePaRD: in 2017, CR: at time of diagnosis.

Abbreviation: AoR: area of residence.

**FIGURE 2:**
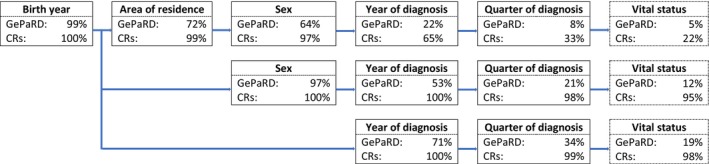
Proportion of non‐unique quasi‐identifier combinations with increasing number of variables used, separately within GePaRD (*N* = 641) and within CRs (*N* = 42 817).

Figure 2 shows the proportion of the non‐unique quasi‐identifier combinations of birth year, AoR, sex, and year and quarter of diagnosis within one single data source, separately for GePaRD (*N* = 641) and CRs (*N* = 42 817). Within GePaRD, 99% of all cases were not uniquely identified by using the birth year. In 8% of all 641 CC diagnoses within GePaRD, there was at least one other GePaRD case with the same birth year, AoR, sex, and year and quarter of diagnosis.

As there are more than 40 different AoRs in Lower Saxony but only 2 in Bremen, the percentage of non‐unique quasi‐identifier combinations differs greatly by CR (Data S1: SM8).

**FIGURE 3:**
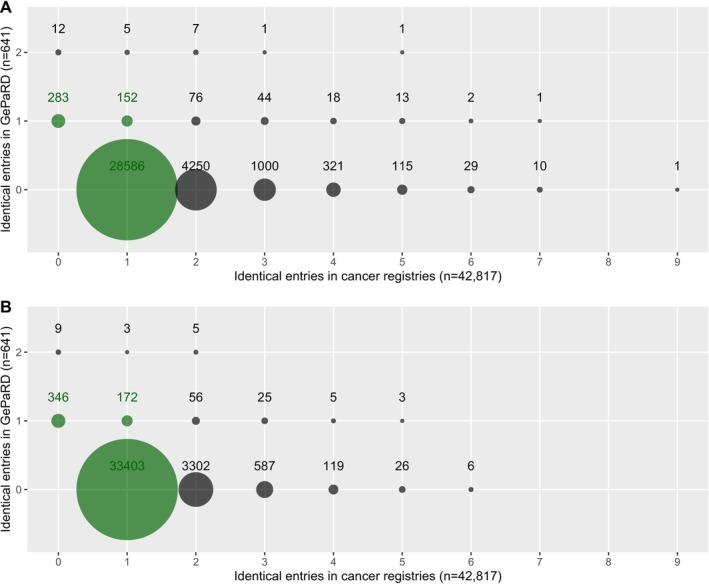
Count of combinations of quasi‐identifiers by number of identical entries from both data sources (A: Birth year, area of residence, sex, and year and quarter of cancer diagnosis, B: Plus vital status). Green: Cases that are uniquely identified when linking both data sources. Grey: Cases that will be part of a linkage cluster with no clear assignment.

Each combination of birth year, AoR, sex, and year and quarter of diagnosis (Figure 3A) might be duplicated in one or both databases, resulting in a cluster of possible links. This decreases the chance of a correct match. There were 152 combinations with a single entry in GePaRD that also appeared only once in the CRs, resulting in an unambiguous link. One combination appeared twice in GePaRD and five times in CRs, causing many ambiguous links. Adding vital status to the quasi‐identifiers resolved some identical matches, but the majority of the duplicates remained (Figure 3B).

## 4 Discussion

We applied different informed classical and ML methods to link German claims and CR data and compared these in terms of linkage quality. We found that an algorithm based on gradient boosting performed best with this real‐world data but was only able to link correctly in about 80% of cases. The observed failure is mainly caused by two aspects. First, information in quasi‐identifiers such as DoDx and ICD codes differs between databases. Second, the capability of the quasi‐identifiers used to substitute a unique identifier in real‐world data remains low, with 8% of not uniquely identifiable patients within the investigated study population of GePaRD.

Informed algorithms lead to the utilization of the maximum sample size and increased representativeness and coverage. For example, in GePaRD, one could use a sub‐sample with available unique identifier to derive a linkage algorithm and transfer the algorithm to the remaining database. Similarly, informed algorithms are useful in many other cases, for example, when linking the National Health Interview Survey and the National Death Index in a US study [25]. In this survey, linkage methods were limited to less precise variables since there have been adjustments both to reduce respondent burden and due to increasing non‐response. Furthermore, individuals from specific subpopulations might have higher risks for mismatches (e.g., due to differences in proportions of missing insurance number). This leads to differential linkage and subsequent selection bias [26, 27, 28]. Expanding these algorithms based on quasi‐identifiers could reduce these problems.

Linking GePaRD and CRs illustrates a common problem in complex real‐world situations when complementary data sources are linked. Those data sources often have different structural designs or different purposes for data collection, which leads to data inconsistencies and differing definitions of variables. In this study, there are discrepancies in the vital status due to a time lag between the updates in the two data sources (Data S1: SM9): some cases that are still alive according to GePaRD have already died according to the more recent information in CRs. The meaning differs for DoDx and ICD10 code between data sources: the DoDx for inpatient cases in GePaRD is the date of admission of the hospitalization that led to the diagnosis. In CRs, the DoDx is the date the case was reported to the CRs. The results of further examinations can still change the ICD code at the time of initial diagnosis (as reported in GePaRD) up to the time of reporting to the CRs. Furthermore, these data sources overlap only partly so that the linkable subset is not identifiable. That is a problem most studies linking populations from different real‐world data sources face [5, 29]. In addition, as both the AoR and the SHI may change over time (and therefore inclusion criteria for CRs and GePaRD), data on some patients, and therefore links, were censored. A solution to these problems would only be possible through the standardization of inclusion criteria and variable definitions. However, this cannot be implemented as the two data sources fulfill different purposes and are fed with data from very different sources (claims vs. doctor's reports).

Considerations when planning a linkage based on quasi‐identifiers should include aspects such as inclusion criteria, data quality, and informativeness of quasi‐identifiers, for example, unique identification via quasi‐identifiers of patients within each data set. In this study, only 12 out of 278 patients with only an outpatient diagnosis of CC according to GePaRD were reported to the CR, which led to their exclusion. A stricter definition of an outpatient diagnosis, for example, when only multiple‐coded diagnoses are considered, might improve linkage results. The investigated data sets have a high data quality, which was confirmed by the few inconsistencies between individual data in CRs and GePaRD. However, using the five common quasi‐identifiers birth year, AoR, sex, and year and quarter of diagnosis, 8% of patients within GePaRD and 33% within CRs were still not uniquely identified.

Two limitations hinder further improvement of the linkage algorithms. First, there is a lack of complete information. No further common potential quasi‐identifiers were available in both databases. Additionally, for data protection reasons, the CRs of Lower Saxony provided only a random sample, which makes it impossible to estimate an appropriate prediction model for not only having cancer but also having reported cancer to a specific CR. Such a prediction might reduce the number of false matches. That means, due to the lack of information, we were unable to check for differential linkage, for example, based on the burden of diseases or personal characteristics, such as migrant status or relocation behavior. The second limitation is the small sample size, which limits the validity of the fitted models. Furthermore, when interpreting the results, it should be kept in mind that overfitting could be present, as the implemented precautionary measures (e.g., subsample selection for gradient boosting) only partially protect against it. Nevertheless, as precision and recall are still considered to be too low for some studies, such linkage results should only be applied with caution.

In summary, the linkage of German claims and CR data based on quasi‐identifiers results in insufficient linkage quality since subjects cannot be uniquely identified. In general, a thorough investigation of quasi‐identifiers and an evaluation of linkage algorithms are recommended to improve the data quality of linked data sets and therefore reduce bias in subsequent analyses. Since the optimal choice of both the algorithm and quasi‐identifiers is highly dependent on the data sources to be linked, multiple informed methods, including gradient boosting, should be considered when true links are available in a subsample.

### 4.1 Plain Language Summary

Clinical trials to investigate the effectiveness and safety of drugs are often expensive, time‐consuming, and sometimes ethically unacceptable. Therefore, claims databases are a useful data source for medical research, but they typically lack information on, for example, tumor stages. Claims data often only contain rough personal information such as birth year or area of residence, which makes a linkage difficult. We analyzed opportunities to find the same person in two different data sources. An exemplary study was conducted on colorectal cancer in users of anti‐diabetic drugs. We used data from German cancer registries and health insurance claims data from the German Pharmacoepidemiological Research Database (GePaRD). Using the common personal information, 33% of the patients could not be uniquely identified within the cancer registry data. For the smaller group of patients from GePaRD, 8% were still not uniquely identifiable. Therefore, linking data sets is only advisable when more detailed personal information is available. Testing for the uniqueness of patients within each data set is beneficial to test the possibilities of combining two data sets using rough personal information. If a unique ID is available in parts of the data, researchers should use this knowledge to develop linking algorithms for the entire data sets.

## Ethics Statement

In Germany, the utilization of health insurance data for scientific research is regulated by the Code of Social Law. All involved health insurance providers as well as the German Federal Office for Social Security and the Senator for Health, Women and Consumer Protection in Bremen as their responsible authorities approved the use of GePaRD data for this study. Informed consent for studies based on claims data is required by law unless obtaining consent appears unacceptable and would bias results, which was the case in this study. According to the Ethics Committee of the University of Bremen, studies based on GePaRD are exempt from institutional review board review. The use of cancer registry data is regulated by the respective regional version of the Cancer Detection and Registration Law. The Senator for Health, Women, and Consumer Protection in Bremen and the Lower Saxony Ministry for Social Affairs, Labour, Health and Equality approved the use of cancer registry data for this study.

## Conflicts of Interest

The authors declare no conflicts of interest.

## Supporting information


Data S1.
