# Supplementary material for: Exploring the Complexity of Real‐World Health Data Record Linkage—An Exemplary Study Linking Cancer Registry and Claims Data
Source: Pharmacoepidemiol Drug Saf. 2025 Mar 25;34(4):e70120. doi: 10.1002/pds.70120 (PMC11934838; doi:10.1002/pds.70120)
Supplement: Supplementary file 1 — Data S1. [file PDS-34-e70120-s001.docx]

# Exploring the complexity of real-world health data record linkage - An exemplary study linking cancer registry and claims data

## SM1 General information about study population

Table 1 Key measures for age and sex of study population

|  | Minimum age | Mean age | Maximum age | Standard deviation age | Proportion of male |
| --- | --- | --- | --- | --- | --- |
| Cancer registries (CRs) | 10 | 72.11 | 105 | 12.22 | 52% |
| GePaRD | 37 | 71.26 | 98 | 10.02 | 52% |

Table 2 Distribution of year of cancer diagnosis in study population

| Year of diagnosis | 2004 | 2005 | 2006 | 2007 | 2008 | 2009 | 2010 | 2011 | 2012 | 2013 | 2014 | 2015 |
| --- | --- | --- | --- | --- | --- | --- | --- | --- | --- | --- | --- | --- |
| GePaRD | 17% | 10% | 4% | 9% | 7% | 9% | 7% | 8% | 8% | 8% | 6% | 6% |
| CRs | - | 10% | 9% | 10% | 10% | 9% | 9% | 9% | 9% | 9% | 8% | 9% |

Table 3 Distribution of quarter of cancer diagnosis in study population

| Quarter of diagnosis | 1 | 2 | 3 | 4 |
| --- | --- | --- | --- | --- |
| GePaRD | 34% | 20% | 21% | 25% |
| CRs | 25% | 26% | 26% | 23% |

Table 4 Number of cases per diagnosis in cancer registries (CRs), GePaRD and in both data sources (gold standard links)

| Catchment area of CR | CC cases in CRs (N) | Patients from antidiabetic cohort in GePaRD (N) | | | | Patients from antidiabetic cohort in GePaRD and CC cases in CRs (gold standard links) (N) | | | | |
| --- | --- | --- | --- | --- | --- | --- | --- | --- | --- | --- |
|  |  | Total | CC cases in GePaRD | | | Total | CC cases in GePaRD | | | Cases without diagnosis of CC in GePaRD |
|  |  |  | Total | Inpatient diagnosis | Outpatient  diagnosis |  | Total | Inpatient  diagnosis | Outpatient  diagnosis |  |
| Bremen | 6.054 | 12.483 | 405 | 237 | 168 | 131 | 123 | 119 | 4 | 8 |
| Lower Saxony | 36.763 | 7.451 | 236 | 126 | 110 | 79 | 76 | 70 | 6 | 3 |
| Total | 42.817 | 19.934 | 641 | 363 | 278 | 210 | 199 | 189 | 10 | 11 |

CRs: cancer registries, CC: colorectal cancer, GePaRD: German Pharmacoepidemiological Research Database

## SM2 Structural differences between cancer registries for dates of diagnosis


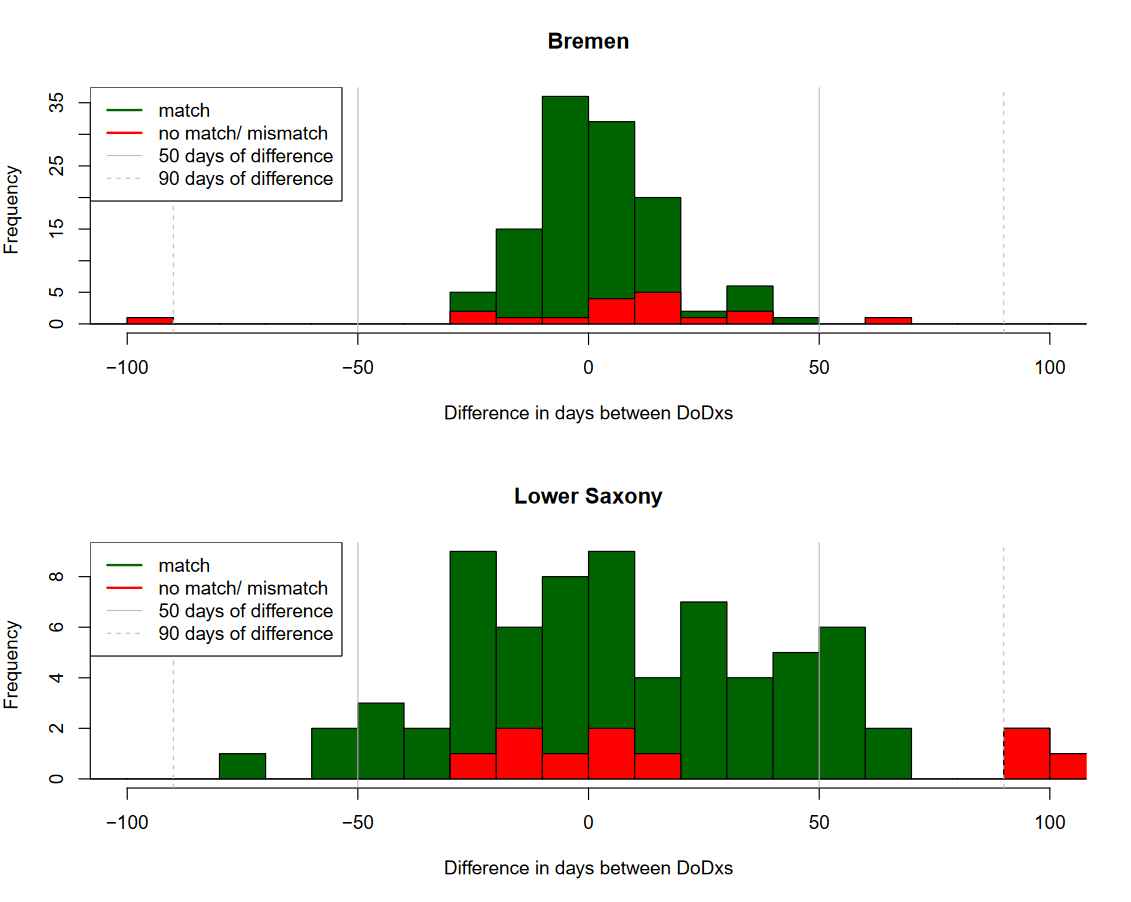


Figure 1 Differences in days between dates of diagnoses (DoDxs) in GePaRD and cancer registriess in Bremen and Lower Saxony for gold standard links according to UDA match status

Figure 2 shows the difference in days between the DoDxs for all gold standard matches to validate the use of a 90 days threshold in the UDA. Green indicates a gold standard match which was found by the UDA. Red indicates a gold standard match which was not identified by the UDA. Gold standard matches in Bremen have a lower range of differences in days between DODxs. Matches with a difference above 50 are not correctly identified by the UDA. In Lower Saxony, there are correctly identified matches with a difference of days between DoDxs of more than 50 but less than 90 days. Therefore, the threshold could be reduced for Bremen but not for Lower Saxony.

## SM3 Parameters used in grid search

Table 5 Parameters used in grid search

| Method | R package | Probability threshold | Parameter (function) | From | To | By | Value chosen |
| --- | --- | --- | --- | --- | --- | --- | --- |
| Logistic regression | stats  version 4.3.0 | 0.1 | - | - | - | - | - |
| Random Forest | ranger  version 0.15.1 | 0.05 | Num.trees (ranger) | 500 | 2500 | 500 | 500 |
|  |  |  | Mtry (ranger) | 1 | 5 | 1 | 5 |
| Gradient boosting | xgboost  version 1.7.5.1 | 0.25 | Max_depth (xgboost) | 3 | 10 | 1 | 5 |
|  |  |  | Max_delta (xgboost) | 1 | 10 | 1 | 8 |
|  |  |  | Nrounds (xgboost) | 500 | 2500 | 500 | 1500 |
| Neural network | torch  version 0.11.0 | 0.0 | Neurons in first hidden layer | 20 | 35 | 5 | 20 |
|  |  |  | Neurons in second hidden layer | 20 | 35 | 5 | 30 |
|  |  |  | Epoch | 10 | 26 | 2 | 18 |
|  |  |  | Learning rate | 0.1 | 0.4 | 0.05 | 0.2 |

R version 4.3.0

Table 5 shows parameters used in grid search to find the best set of parameters. A grid search compares every combination of parameter values according to a certain measure (in this case precision). Parameter values start with the value in column 'From', in steps according to column 'By' and end with the value in 'To'. The best value according to grid search can be found in column ‘Value chosen’. The parameters to be tuned are a standard selection, but are limited due to the scope of the study.

## SM4 Second chance effect

In this study, each GePaRD and each CRs case can only be linked once. Some GePaRD cases lose their link due to one CRs case being linked multiple times. Those might be linked again with the second best fit from CRs. The resulting effect is minor.

Table 6 Linkage results without second best

|  |  | Logistic regression | Gradient boosting | Random forest | Neural network |
| --- | --- | --- | --- | --- | --- |
| True positives |  | 155 | 164 | 147 | 94 |
| Mismatches |  | *90 (-1)* | *47 (-2)* | 102 | *184 (-8)* |
| Total links |  | 245 | 211 | 249 | 278 |
| Precision |  | 63% | **78%** *(+1%pt.)* | 59% | *34% (+1%pt.)* |
| Recall |  | 76% | **81%** | 72% | 46% |
| F*-measure |  | 53% | **66%** *(+1%pt.)* | 48% | 24% |

## SM5 Overview of used methods

### Uninformed deterministic algorithm (UDA)

The algorithm by Kollhorst (2022) runs as follows:

1. merges all inpatient diagnoses from GePaRD with every diagnosis from CRs within 90 days and with accordant area of residence, sex, and birth year,
2. classifies links as ‘match on ICD10 (four-digits)’, ‘match on ICD10 (three-digits)’, or ‘no match on ICD10’ and keeps only links from the best category per GePaRD case,
3. chooses the CR case with the closest date of diagnosis,
4. chooses one random case per GePaRD case (if there are multiple), and
5. chooses one random case per CR case (if there are multiple).

### Informed deterministic algorithm (IDA)

The updated algorithm (adjustments in *italics*):

1. merges all inpatient diagnoses from GePaRD with every diagnosis from CRs within *90 days (Lower Saxony) / 50 days (Bremen)* and with accordant area of residence, sex, and birth year,
2. classifies links as ‘match on ICD10 (four-digits)’, ‘match on ICD10 (three-digits)’, or ‘no match on ICD10’ and keeps only links from the best category per GePaRD case,
3. chooses the CR case with the closest date of diagnosis,
4. *for deceased patients according to GePaRD, prefer cases also deceased according to CRs over those cases still alive according to CRs,*
5. chooses one random case per GePaRD case (if there are multiple), and
6. chooses one random case per CR case (if there are multiple).

### Naïve deterministic 1:1 matching

This algorithm only confirms links with matching link variables where both observations concerned do not belong to any other possible link (i.e. have identical values in all link variables with other persons).

### Logistic regression

Binary logistic can be applied as classification algorithm modeling a probability of an observation belonging to one out of two groups. Observations with a prediction above a threshold are assumed to belong to that group.

### Gradient boosting

Gradient boosting is a robust tree-based method. Decision trees are grown iteratively and each tree is weighted to reduce the prior mistakes according to a problem-specific loss function. This method needs little tuning effort and performs well on tabular data. A regularization term in the loss function shall reduce overfitting. A disadvantage is the binary split of the decision tree – complicated patterns are hard to model.

### Random Forests

Random forests are another ensemble method based on decision trees. Unlike gradient boosting the trees are independently distributed. Each tree is built using a random subsample of observations and covariates, thereby reducing potential overfitting. Random forests also suffer from the binary split of the decision trees.

### Neural networks

The machine learning method neural networks are a black box method inspired by the architecture of (human) brains. It consists of several layers. Each layer consists of neurons which get activated by the input. Certain combinations of activated neurons lead to the outputs. Neural networks are effective especially in unstructured data as images or audio by finding hidden and complex patterns. The algorithms used to train the neural network depends on the task. In general, high tuning efforts are necessary for good results.

### Probabilistic record linkage

Two kinds of probability are used to calculate a match weight. The m-probability gives the probability that related entries do not match due to e.g. transmission errors or changes between data collections. The u-probability gives the probability for a match by chance. These probabilities differ depending on the covariate and survey - AoR, for example, only had two values in the Bremen data and therefor high chances of matching by chance. Each covariable is assigned a match weight calculated from m-, u-probability and matching status. The sum of those match weights per covariate is the final match weight for the link. Links are then grouped into three categories according to the match weight: no link (below lower threshold), clerical check necessary (between lower and upper threshold), and confirmed link (above upper threshold). If a clerical check is not possible due to privacy or costs, the lower and upper threshold are identical. A disadvantage of probabilistic record linkage is the need to know the true match status to calculate the correct m- and u-probabilities. Alternatively, m- and u-probabilities are selected based on experience.

## SM6 Overlap of patients in GePaRD and CRs and distribution of reasons of exclusions


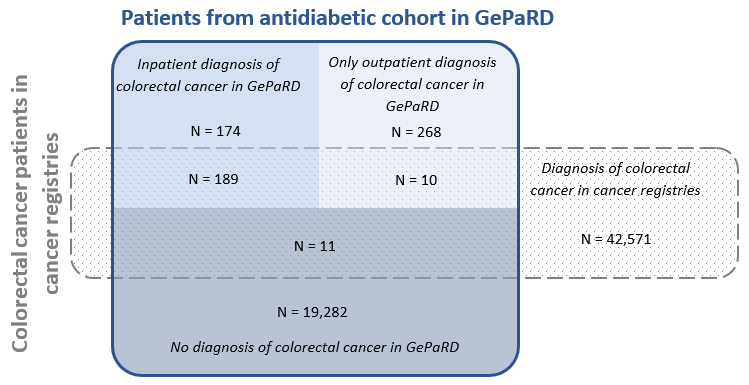


Figure 1 Overlap (gold standard links) of patients in GePaRD and CRs and distribution of reasons of exclusions

The antidiabetic cohort in GePaRD consists of three subgroups – those that had at least one inpatient diagnosis of CC in GePaRD (N = 363), those that had at least one outpatient but no inpatient diagnosis of CC in GePaRD (N = 278) and lastly those with no diagnosis of CC in GePaRD (N = 19,293). The cohort partly overlaps with the group of colorectal cancer patients in the two CRs according to the gold standard. The overlap consists of 189 patients with inpatient diagnosis, 10 with outpatient diagnosis and 11 patients with no diagnosis of colorectal cancer in GePaRD. Out of the antidiabetic cohort in GePaRD, 19,282 patients have no CC report from GePaRD and no gold standard link. Interestingly, 11 cases have no diagnosis of CC in GePaRD but appear in the CRs as having CC according to the gold standard. Those cases might have changed SHIs before their CC diagnosis. From the cohort, 174 patients have an inpatient diagnosis of CC in GePaRD but could not be found in CRs data by the gold standard. E.g. those patients might have moved from the catchment area of one CR to that of another. Out of the CRs data, 42,571 patients have no gold standard link in GePaRD.

## SM7 Detailed results of the probabilistic record linkage

Usually, in probabilistic record linkage two thresholds are used. All weights above the upper threshold indicate the link is confirmed by the algorithm. All weights between lower and upper threshold indicate the corresponding link should be subjected to a clerical review. In the case of data from GePaRD and CRs, a clerical review is not possible for data protection reasons. Therefore, both lower and upper threshold are identical. Table 7 shows that 5 links have a match weight of 31.85, one link a match weight of 31.69 and around 1,500 links a match weight of 17.06. Therefore, a threshold larger than 17.06 and lower than 31.69 results in 6 links. Reducing the threshold below 17.06 results in the acceptance of around 1,500 additional potential links – for only 363 inpatient diagnosis from GePaRD. Usually the link with the highest match weight per GePaRD case is chosen. In this case, the identical match weight of 17.06 prevents this mechanism. Apparently, there are too little information available to properly distinguish between links and non-links.

Table 7 Distribution of match weights for probabilistic linkage

| Weight | 16.84 | 16.90 | 17.00 | 17.06 | 31.69 | 31.85 |
| --- | --- | --- | --- | --- | --- | --- |
| Number of potential links | 27 | 1260 | 35 | 1497 | 1 | 5 |

## SM8 Proportion of non-unique quasi-identifier combinations per cancer registry


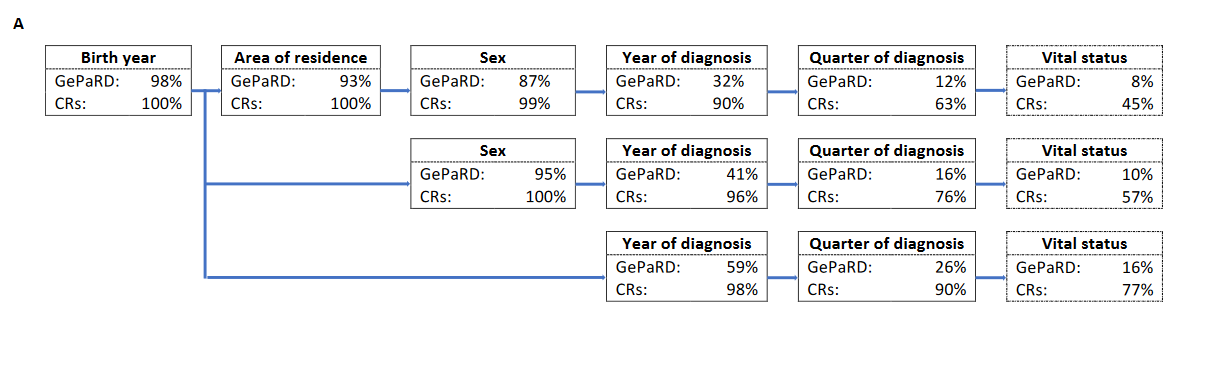

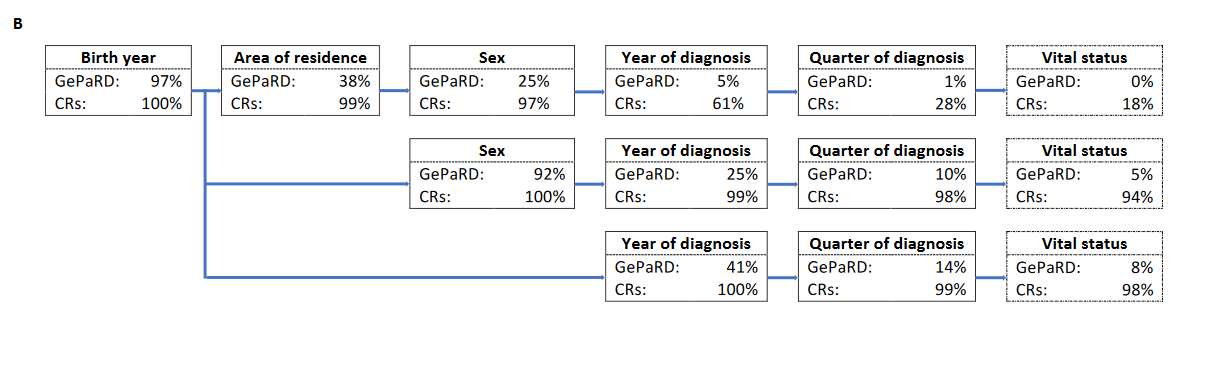


Figure 3 Proportion of non-unique quasi-identifier combinations with increasing number of variables used in Bremen (A, GePaRD: N=405, cancer registries (CRs): N=6,054).) and Lower Saxony (B, GePaRD: N=236, cancer registries (CRs): N=36,763).

## SM9 Time lags of data sources

Table 8 Time of data delivery and latest death for GePaRD and CRs

| Source | Delivery of Data | Latest date of death |
| --- | --- | --- |
| GePard: First health insurance company | 2017 | 2017 |
| GePard: Second health insurance company | <= 2017 | 2016 |
| CR of Bremen | 2020 | Unknown |
| CR of Lower Saxony | 2021 | Unknown |

Due to data protection reasons, there is no information on date of death in the available cancer registry data. The only available information on death is “alive”, “dead due to any cancer”, or “dead due to other”. From GePaRD, the latest available date of death is from 2017 (Table 8). Later deaths would be included in subsequent data deliveries which could not be used due to data protection reasons.

Due to the large gap between dates of data delivery and the high percent of patients alive per GePaRD data but deceased per cancer registry data, there seems to be updated information in cancer registries compared to GePaRD.
